# Supplementary material for: Determination of the Elution Capacity of Dalbavancin in Bone Cements: New Alternative for the Treatment of Biofilm-Related Peri-Prosthetic Joint Infections Based on an In Vitro Study
Source: Antibiotics (Basel). 2022 Sep 23;11(10):1300. doi: 10.3390/antibiotics11101300 (PMC9598415; doi:10.3390/antibiotics11101300)
Supplement: Supplementary file 1 [file antibiotics-11-01300-s001.zip › antibiotics-1938769-supplementary.pdf]

Figure S1. Preparation of the antibiotic-loaded bone cements

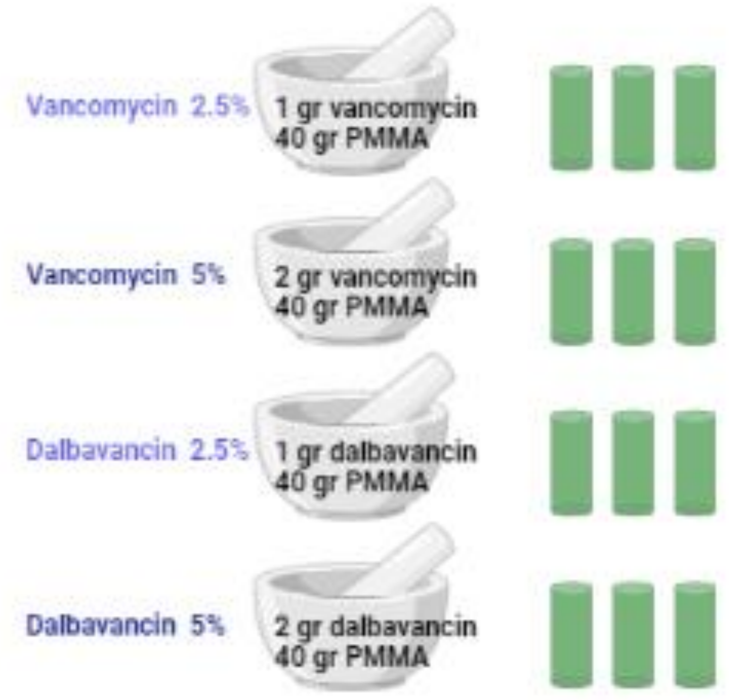

PMMA, Polymethylmethacrylate; **gr**, grams.

Figure S2. Laboratory procedure

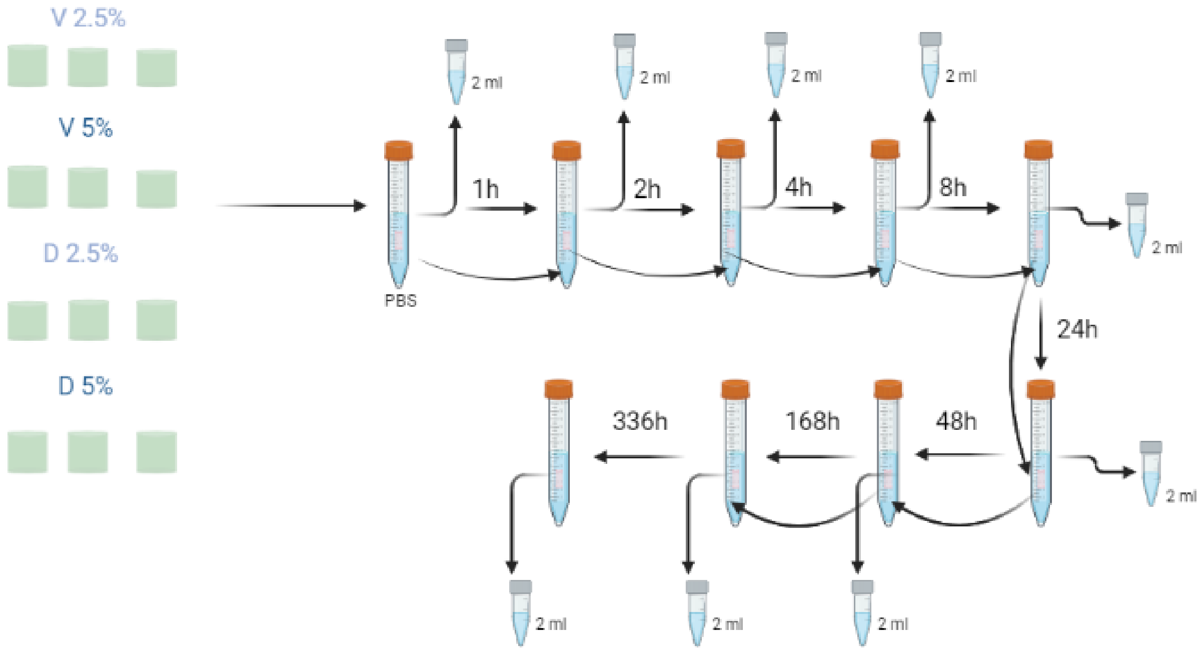

V, vancomycin; **D**, dalbavancin; **h**, hours.
